# Fermitin family homolog 2 (Kindlin-2) affects vascularization during the wound healing process by regulating the Wnt/β-catenin signaling pathway in vascular endothelial cells

**DOI:** 10.1080/21655979.2021.1957526

**Published:** 2021-08-02

**Authors:** Jianghui Ying, Qiang Wang, Lu Lu, Jiaqi Liu, Rong Guo, Hao Hu, Hua Jiang, Fazhi Qi

**Affiliations:** a Department of Plastic Surgery, Zhongshan Hospital, Fudan University, Shanghai, China; b Department of Plastic Surgery, Shanghai East Hospital, School of Medicine, Tongji University, Shanghai, China

**Keywords:** Kindlin-2, wound healing, vascularization, Wnt/β-catenin pathway

## Abstract

Kindlin-2 is a member of the FERM-containing cytoskeletal protein family that regulates cell–matrix interactions. Previous studies have shown that Kindlin-2 recruits focal adhesion proteins and regulates integration by binding to the focal adhesion region of the integrin β-segment. Although Kindlin-2 has been reported to be involved in various skin diseases and many kinds of tumors, its role in the skin wound healing process remains unclear. The aim of the present study was to investigate the role of Kindlin-2 in the regulation of wound healing. The effects of Kindlin-2 on wound healing were studied by a wound healing model, kindlin-2 (±) mice. The effects of Kindlin-2 on cell migration, cellular tube formation, and cell adhesion and spreading were evaluated in human umbilical vein endothelial cells (HUVECs) with downregulated Kindlin-2 expression. We found that the expression of kindlin-2 was elevated in wound healing tissues and that interfering with the expression of Kindlin-2 delayed the wound healing process and reduced neovascularization. We found that the wound healing of kindlin-2 (±) mice was delayed, with a decreased number of new blood vessels. Furthermore, depletion of Kindlin-2 impaired HUVEC spreading, migration and tube formation. Intriguingly, we found that kindlin-2 binds to β-catenin in the Wnt/β-catenin signaling pathway and cooperates with β-catenin to enter the nucleus from the cytoplasm, activating the downstream Wnt/β-catenin signaling pathway. Taken together, these results help to elucidate the mechanism of Kindlin-2 in the regulation of the wound healing process and provide a theoretical basis for further study of wound healing and abnormal healing.

## Introduction

Skin wound healing is the process of repairing damaged tissue, which can be injured by an external force [1]. According to statistics, approximately 1% of the world’s population is affected by persistent wounds, and approximately 5% of medical expenses are spent on wound repair [2]. These conditions cause major physical and mental distress to the patient. To study the mechanism of the repair process, researchers have widely used transgenic mouse models in recent years to help define the roles of many key molecules [3]. Transgenic mice are genetically modified by gene recombination in mouse embryonic stem cells; that is, the targeted genes in embryonic stem cells are changed, and then, the ‘modified’ embryonic stem cells are implanted into the early embryos of mice to generate chimeric mice [4]. This method is a pioneering method in the field of biomedicine [5]. Moreover, because human genes have more than 90% similarity with mouse genes, it is theoretically possible to study diseases caused by human genetic factors such as inflammation and wound healing [6]. For example, IL-6 -/- mice exhibited significantly delayed skin wound healing compared with wild-type animals [7]. CXCR2-deficient transgenic mice displayed defective neutrophil recruitment, resulting in significant delays in epithelialization and decreased neovascularization [8]. The molecules found in transgenic mouse models can be used as targets for the development of drugs for the treatment of wounds.

Angiogenesis is the process of establishing new blood vessels and is a fundamental physiological process that provides oxygen and nutrients for tissue repair and chronic inflammation [9]. Neovascularization begins with vascular endothelial cells. Most of the new blood vessels in the wound form new vascular buds, and others develop by recruiting bone marrow-derived endothelial progenitor cells from the systemic circulation [10]. Normal angiogenesis is an important part of repairing wounds and tissue remodeling. Abnormal or excessive angiogenesis will lead to diseases such as diabetic foot and diabetic retinopathy [11].

The kindlin family is a family of adaptor proteins with highly conserved FERM domains. These proteins can be used as scaffolds to connect the cell membrane and cytoskeleton [12]. As a typical member of the FERM protein family, kindlins consist of F1, F2 and F3 subdomains, with an N-terminal F0 subdomain. A remarkable feature of kindlins is the insertion of pleckstrin homology (PH) into the F2 subdomain [13]. The kindlin family can be divided into three subtypes: kindlin-1, kindlin-2 and kindlin-3. Kindlin-2 is expressed in early embryos and widely distributed in all tissues and cells. Kindlin-1 and kindlin-3 are expressed in the epidermis and blood cells, respectively [14–16]. Previous studies have shown that kindlin-2 can recruit focal adhesion proteins and regulate integration by binding to the focal adhesion region of the integrin β-segment. The abnormal expression of Kindlin-2 has been confirmed to be closely related to various skin diseases and many kinds of tumors, but its role in the wound healing process have hardly been reported [17]. In this study, we used knockout mouse models for the first time to study the impact of a single gene on skin wound healing. We addressed the expression and functions of kindlin-2 in HUVECs in vitro and in the skin in situ and showed that kindlin-2 regulates adhesion and directed migration of HUVECs by mediating the Wnt/β-catenin signaling pathway.

In the present study, we hypothesized that Kindlin-2 is involved in the progression of wound healing by activating the downstream Wnt/β-catenin signaling pathway. To prove this hypothesis, we detected the expression levels of Kindlin-2 during the wound healing process and evaluated its effects on wound healing in a wound healing model of kindlin-2 (±) mice. The effects of Kindlin-2 on cell migration, cellular tube formation, and cell adhesion and spreading were evaluated in HUVECs with downregulated Kindlin-2 expression. Additionally, the relationship between Kindlin-2 and β-catenin in HUVECs was investigated.

## Materials and methods

### Ethical approval of the study protocol

All animal experiments were carried out according to the recommendations of the Guide for the Care and Use of Laboratory Animals, National Institutes of Health (NIH) and conformed to the guidelines of the Declaration of Helsinki. The study was approved by the Ethics Committee of Zhongshan Hospital, which is affiliated with Fudan University.

### Experimental groups

Mice were bred in an SPF-grade experimental animal room. All surgical experiments were carried out using sodium pentobarbital anesthesia. Male C57BL/6 mice used to detect the expression of kindlin-2 during wound healing were 8–9 weeks old and were purchased from SLAC Laboratory Animals (Shanghai, China). Kindlin-2 (±) mice constructed by Cas9 technology were purchased from Model Animal Research Center (Nanjing, China). The sgRNA sequence is 5ʹ-GTTTGTGCTGCGGGTGAAC- 3ʹ. When the chimeric mice grew, there were both modified and unmodified genes in their bodies. If the germ cells of some mice are ‘modified’, they will produce mice with completely ‘modified’ genes [18]. Mice were divided randomly into two groups: the WT group and the Kindlin-2 (±) group.

### Mouse wound model

The mouse wound model was created as previously described [19], with 36 mice included in the study. Following the induction of anesthesia, the dorsal skin was shaved and disinfected using 75% ethanol. Skin wounds were created using a 6-mm skin punch and scissors. Five days after wounding, healing tissues were removed from some mice for further experiments, including immunofluorescence and Western blot, and the remainders of the mice were used to evaluate wound healing and blood vessel permeability. After four weeks, the mice were euthanized by cervical dislocation under anesthesia.

### RT-PCR

Real-time quantitative PCR (qPCR) was performed using the QuantStudio™ 3 Real-Time PCR System (ABI, USA), and expression values were normalized to that of the housekeeping gene GAPDH. The primers used for amplification were as follows: for kindlin-2, F: 5ʹ-GAACAAGCAGATAACAGCGAGA-3ʹ and R: 5ʹ-TGGAAC CTTGCAATGAAGTG-3ʹ.

### Western blot

The mouse wound tissues were weighed and lysed in RIPA buffer with inhibitors and quantified by BAC as previously described [20]. Then, the lysates were subjected to SDS-PAGE followed by transfer onto a PVDF membrane. The antibodies used were rabbit monoclonal antibodies against Kindlin-2 (Sigma-Aldrich) and GAPDH (Cell Signaling Technology). The blots were visualized using enhanced chemiluminescence (ECL) reagent. ImageJ software (National Institutes of Health, Bethesda, MD, USA) was used to analyze blot density.

### Immunofluorescence

Mouse skin tissues were sectioned using a cryostat microtome in a freezing chamber. First, the sections were permeabilized with 0.1% Triton X-100 for 30 min at room temperature. The sections were incubated in blocking solution for 60 min at room temperature. Immunostaining was performed using primary antibodies that included a rat anti-CD31 antibody (Santa Cruz) and a rabbit anti-Kindlin-2 antibody (Sigma-Aldrich) with overnight incubation at 4°C, followed by incubation with the fluorescein isothiocyanate (FITC)-conjugated secondary antibody for 1 h at room temperature. Six samples were selected from each group of mice, and four image views were captured from each sample by immunofluorescence microscopy.

### Macroscopic analysis of the wound area

The wound area was photographed using a digital EOS40D camera (Canon, Tokyo, Japan) at one, three, six, and eight days post-wounding. The wound area was calculated using ImageJ software.

### Evaluation of blood vessel permeability

Vascular permeability was measured as previously described [19]. Two weeks after the mouse skin wounds had healed, 100 μL of Evans Blue dye (EBD) (Sigma-Aldrich) was injected intravenously, and mustard oil was used on the dorsal skin. Thirty minutes after injection with EBD, skin samples of similar size were removed, weighed, and photographed. Then, the EBD was extracted with 1 mL of formamide overnight at 60°C using constant agitation. The amount of EBD extracted was measured using a spectrophotometer at 610 nm. The skin was dried for 72 h at 60°C and weighed, and the absorbance was measured.

## Statistical analysis

### Cell culture

The human HUVEC line was obtained from the American Type Culture Collection (ATCC, USA). Cells were cultured in DMEM (HyClone, USA) supplemented with 10% FBS (HyClone, USA). Cells were grown at 37°C in humidified conditions with 5% CO2.

### Cell proliferation assay

The cells in each group were digested by trypsin and made into a cell suspension. The cells in each group were seeded in 96-well plates at 103 cells/well, with 3 wells in each group. After 24 hours, 48 hours, 72 hours, 96 hours and 120 hours, 10 µl of CCK-8 reagent and 90 µl of culture medium were added, and the culture was continued for 2 hours. The OD value at 450 nm was measured and analyzed statistically.

### Cell spreading and adhesion assays

The cells in each group were digested by trypsin and made into a cell suspension.

A cell climbing plate was inserted into the 6-well plate, and the cell suspension was inoculated. Cells were seeded in each well, and 3 wells were set for each group. After culture in a 5% CO2 incubator at 37°C for 4 hours, the supernatant was removed and fixed with 4% paraformaldehyde for 15 minutes. The fixed cells were dehydrated with 30%, 50%, 70%, 85%, 90% and 100% ethanol. Each stage lasted for 30 minutes. The cells were dried in natural air, and the surface of the cells was treated by spraying gold 60 s, 20 kV). The adhesion of HUVECs was observed by field emission scanning electron microscopy.

For cell adhesion assays, the fixed cells were incubated with DAPI for 5 min in the dark, and images were taken by fluorescence microscopy and quantified by measuring the optical density.

### Cell migration assay

After trypsin digestion, the cells were centrifuged. Then, the supernatant was removed, and the cells were counted after serum-free medium was added. Transwell chambers (8.0 μM) were placed in a 24-well plate with 600 µl of DMED containing 10% FBS serum in the lower layer. Then, cells (1 × 104) were added to the upper layer and cultured in an incubator for 12 hours. After that, the cells were removed by gently wiping the upper chamber with a cotton swab. Polymethyl alcohol was used for fixation for 20 min, followed by dying with crystal violet for 30 minutes, rinsing with distilled water and drying naturally.

### Wound healing assay

The cells were digested by trypsin, and then, the suspension was made and inoculated in a 6-well plate. Each group had 3 wells. The cells were cultured in a 5% CO2 and 37°C incubator until 90% confluence. The supernatant was aspirated, and the yellow pipette head was held vertically to make a cross-cell scratch. PBS was used to wash and scrape the cell debris, and serum-free DMEM was added. After photos were taken, the cells were cultured in a 5% CO2 incubator at 37°C. The cells were photographed under a microscope at 24 hours and 48 hours to observe the degree of cell fusion.

### Cell tube formation

One day before the experiment, the matrix adhesive, pipette head and 96-well plate were predissolved or precooled in the refrigerator at 4°C. Fifty microliters of matrix adhesive was incubated on ice and evenly spread on 96-well plates. The 96-well plates were placed in the cell incubator for 30 minutes, and then, the gel was allowed to set. When HUVECs grew to 80–90%, they were digested by trypsin and made into a suspension. The 96-well plates covered with matrix adhesive were filled with cells, and 1 × 104 cells per well were added to DMEM containing 10% FBS. The cells were cultured in a 5% CO2 incubator at 37°C for 4 hours. The cells were observed and pictures were taken under the microscope.

### Statistical analysis

Data are presented as the mean ± standard error of the mean (SEM). Statistical analysis was performed using a Kolmogorov-Smirnov normality test. Between-group comparison of means was performed by one-way analysis of variance (ANOVA). P < 0.05 was considered statistically significant.

## Results

### Kindlin-2 expression is elevated during wound healing

**Figure 1.**
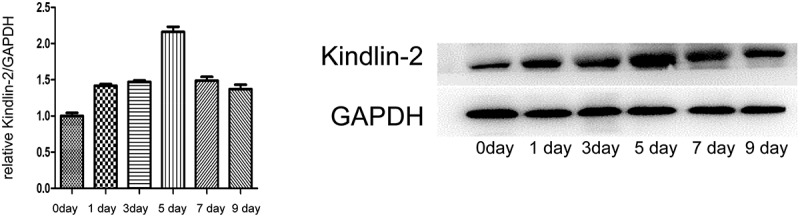
**Kindlin-2 expression is elevated during wound healing**. a: Real-time PRC was used to detect the expression of kindlin-2 at 1, 3, 5, 7 and 9 days after injury, and GAPDH was used as an internal reference for quantification of relative expression. Compared with the relative expression of kindlin-2 on day 0, multiple relationships were obtained (P < 0.05). b: The dynamic expression of kindlin-2 protein was detected by Western blots, and GAPDH was used as an internal reference

Kindlin-2 has been reported to be involved in various skin diseases and many kinds of tumors; however, its role in the skin wound healing process is unclear. In the present study, we hypothesized that Kindlin-2 is involved in the progression of wound healing by activating the downstream Wnt/β-catenin signaling pathway. To explore kindlin-2 expression levels in wound healing, we constructed a wound healing model in mice. Real-time PCR and western blotting were used to determine the dynamic expression of Kindlin-2 in the wound healing process. As shown in Figure 1, on the first day after injury, the expression of kindlin-2 began to gradually increase, the RNA and protein expression levels of Kindlin-2 reached its peak on day 5, and the expression abundance was 2.38 times that of normal wounds, after which the expression of Kindlin-2 gradually returned to normal. On the 9th day, the expression level was 1.36 times that in normal wounds.

### Effect of Kindlin-2 on wound healing in kindlin-2 (±) mice

**Figure 2.**
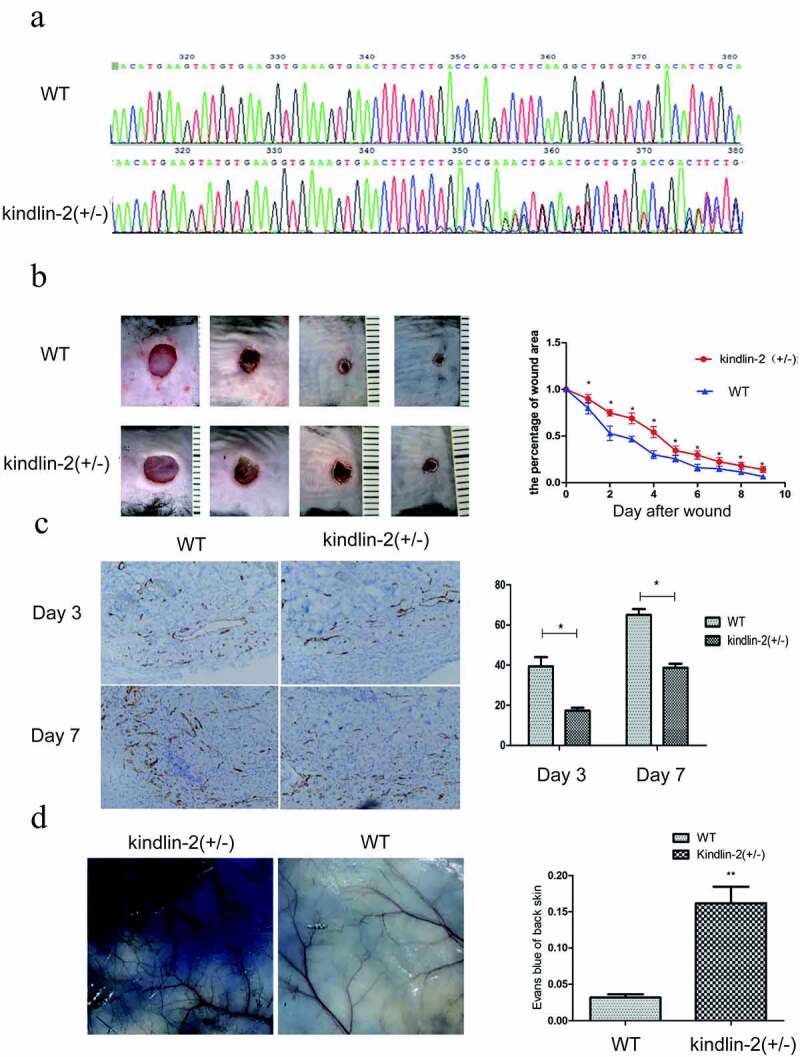
**Effect of Kindlin-2 on wound healing in kindlin-2 (±) mice**. a: Kindlin-2 (±) heterozygosity resulted in a bimodal image 350 bp behind that of normal control mice. b: Kindlin-2 gene knockout prolonged the healing time of mouse back wounds, * indicates P < 0.05. c: Skin wounds on day 3 and day 7 were selected, and CD31 (brown) in vascular endothelial cells was identified by immunohistochemistry. The bar = 100 microns. d. Comparison of the differences between the two groups by calculating the absorbance of Evans Blue through skin exudation

Then, kindlin-2 (±) mice were constructed by Cas9 technology to explore the biological function of kindlin-2 in wound healing (Figure 2(a)), and kindlin-2 (±) mice and normal mice were analyzed by immunohistochemistry, HE staining, Masson staining and other experimental methods. As shown in Figure 2(b), the wound healing process of the kindlin-2 (±) mice was delayed, and the area of residual wounds was significantly larger than that of the normal mice at all stages. As angiogenesis plays an important role in wound healing, we detected the number of new blood vessels by staining CD31. We found that the number of new blood vessels was significantly decreased at day 3 and day 7 (Figure 2(c)). Moreover, the permeability of the neovasculature was increased (Figure 2(d)), as shown by Evans Blue examination. These results further confirmed that Kindlin-2 is involved in angiogenesis during wound healing. Angiogenesis is an indispensable physiological process for wound healing. Normal blood flow can provide sufficient nutrition and tissue metabolic waste for tissue reconstruction. Abnormal angiogenesis will lead to delayed wound healing [21]. Therefore, we investigated the biological function of kindlin-2 in blood vessel cells.

### Downregulation of Kindlin-2 expression inhibits cell adhesion and spreading

**Figure 3.**
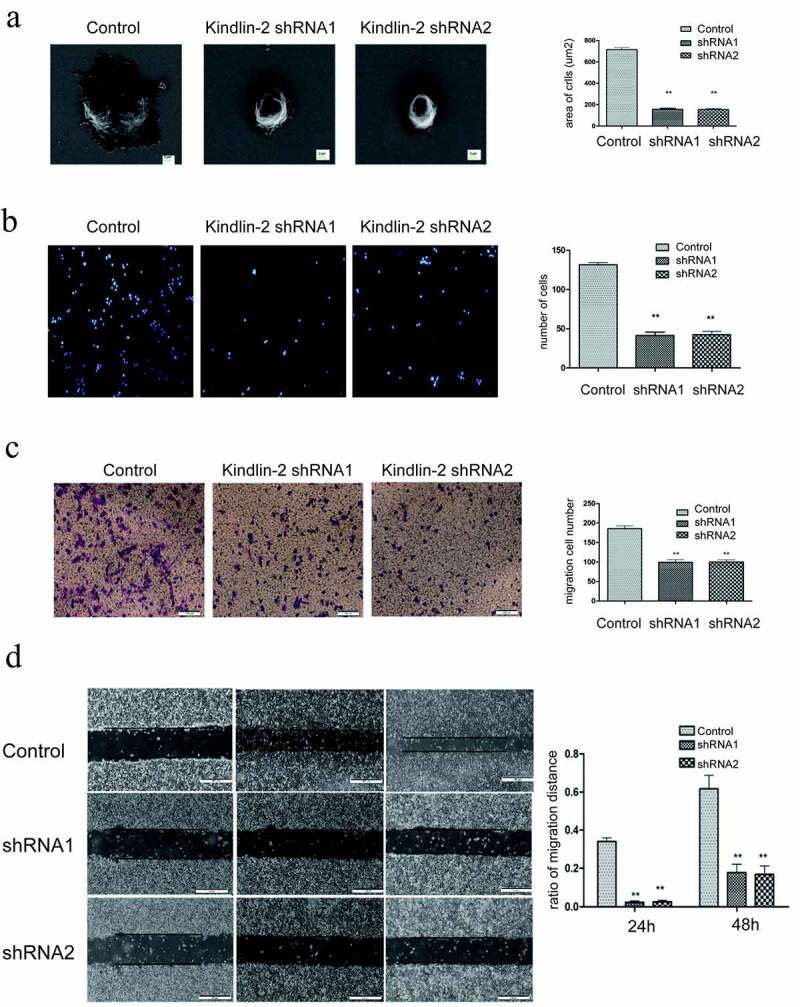
**Down-regulation of Kindlin-2 expression inhibits cell migration, cell adhesion and spreading**. a: Morphological changes in vascular endothelial cells after 4 hours of adherence were observed by field emission electron microscopy. Bar = 2 μm. The spreading area of cells in each group was calculated by ImageJ. b: The adhesion of cells in each group for 4 hours was observed by immunofluorescence. Bar = 50 microns. The adhesion ability of each group was compared by calculating the number of adherent cells. c: Transwell chamber experiments were conducted to observe the migration changes of the control group, Kindlin-2 shRNA1 group and Kindlin-2 shRNA2 group after 12 hours, bar = 200 μm. d: The migration of the control group, Kindlin-2 shRNA1 group and Kindlin-2 shRNA2 group was observed to be 500 μm by the scratch test. The migration ability of each group was compared by calculating the relative migration distance

To further investigate the role of Kindlin-2 in umbilical vein endothelial cells (HUVECs), we transformed Kindlin-2 shRNA into HUVECs by lentivirus (Supplementary Figure 1a&b). Downregulation of Kindlin-2 expression did not affect the proliferation of HUVECs (Supplementary Figure 1d) but significantly decreased the spreading and adhesion of umbilical vein endothelial cells (Figure 3(a and b)). As shown in Figure 3(a), the spreading areas of the downregulation groups were much smaller than those of the control group. The number of adhering cells in the control group was 2.5 times that in the downregulation groups.

### Downregulation of Kindlin-2 expression inhibits cell migration and cellular tube formation

**Figure 4.**
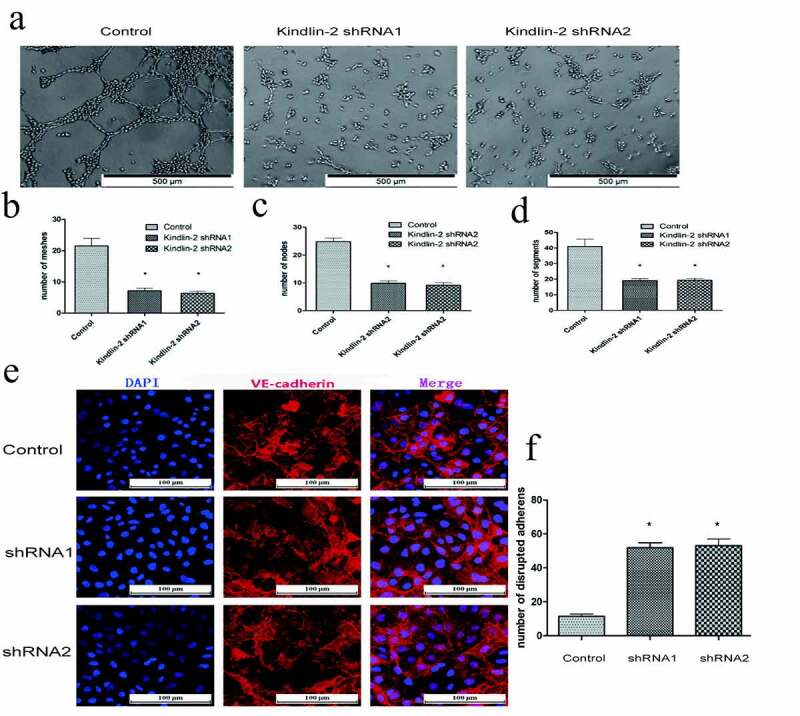
**Down-regulation of Kindlin-2 expression inhibits cellular tube formation**. a: Tube images of the control group, Kindlin-2 shRNA1 group and Kindlin-2 shRNA2 group cultured on matrix glue; bar: 500 μm. b-d: Quantification of the number of small pipe segments, segments and grids. e: The distribution of tight junctions was detected by immunofluorescent VE-cadherin staining

Transwell and wound healing assays showed that downregulation of Kindlin-2 expression inhibited cell motility (Figure 3(c and d)). The number of migrating cells in the control group was approximately 2 times that in the downregulation groups (Figure 3(c)), and the control group also showed faster migration than the downregulation group (Figure 3(d)). Furthermore, tube formation assays showed that knocking down kindlin-2 impaired tube formation in HUVECs (Figure 4(a)). We compared the number of cellular nodes, segments and meshes, and all three numbers in the control group were 2 times greater than those in the experimental group (Figure 4(b,c and d)). Vascular endothelial cell cadherin (VE-cadherin) is a homodimer transmembrane protein that is expressed significantly in vascular endothelial cells [22]. Its cytoplasmic domain binds to a variety of intracellular ligands, including p120 and β-catenin, which are essential for the stability of adhesion and vascular integrity [23]. Therefore, we detected the distribution of tight junctions by immunofluorescent VE-cadherin staining. As shown in Figure 4(e), the number of disrupted adherens junctions was increased in the sh-kindlin-2 groups. A decrease in kindlin-2 expression led to a decrease in endothelial cell migration, adhesion, spreading and tube forming ability and affected angiogenesis. This phenomenon also affected the structure of VE cadherin, destroyed the tight junctions between vascular endothelial cells, and increased the permeability of neovasculature. These results confirmed that Kindlin-2 regulates the biological function of HUVECs.

### Downregulation of Kindlin-2 expression inhibits β-catenin nuclear transport

**Figure 5.**
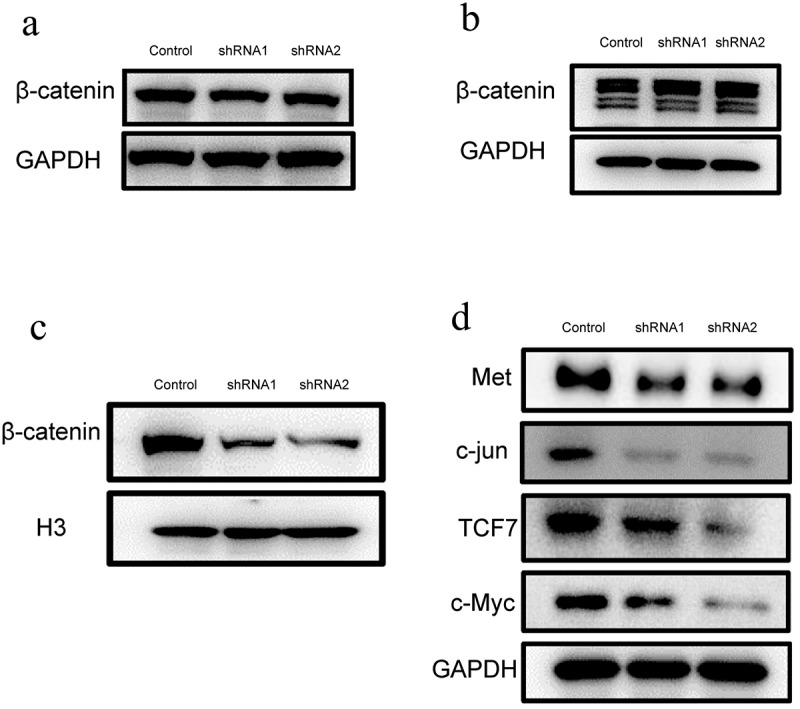
**Down-regulation of Kindlin-2 expression inhibits β-catenin nuclear transport**. a: β-catenin protein expression in endothelial cells (including cytoplasm and nucleus) was detected by Western blots in the control group, kindlin-2 shRNA1 group and kindlin-2 shRNA2 group. b: The expression of β-catenin protein in the cytoplasm was detected by Western blots in the control group, kindlin-2 shRNA1 group and kindlin-2 shRNA2 group. c: The protein expression of β-catenin in the nucleus was detected by Western blots in the control group, kindlin-2 shRNA1 group and kindlin-2 shRNA2 group. d: The protein expression levels of Met, c-Jun, TCF7 and c-Myc in the control group, Kindlin-2 shRNA1 group and Kindlin-2 shRNA2 group were detected by Western blots

The Wnt signaling pathway plays an important role in regulating the physiological functions of a variety of cells, including proliferation, repair and angiogenesis. Thus, we detect the expression of β-catenin. Western blot analysis showed that β-catenin was decreased slightly after Kindlin-2 expression was decreased (Figure 5(a)) but was differentially expressed in the nucleus and cytoplasm. As shown in Figure 5(b), the amount of β-catenin in the cytoplasm increased by 1.5 times in the kindlin-2 downregulation groups compared with the control group. Moreover, the amount of β-catenin in the nucleus was reduced by half in the kindlin-2 downregulation groups compared with the control group (Figure 5(c)). Therefore, the expression of kindlin-2 might influence the Wnt/β-catenin pathway. Furthermore, the expression of downstream proteins, such as Met, c-jun, TCF7 and c-Myc, was significantly inhibited in the kindlin-2 downregulation groups (Figure 5(d)). These results suggest that Kindlin-2 affects the activity of the Wnt/β-catenin signaling pathway by affecting the translocation of a key protein.

In summary, this study demonstrates that Kindlin-2 is highly expressed during wound healing and participates in angiogenesis in the wound healing process. This molecule binds to β-catenin in the Wnt/β-catenin signaling pathway and cooperates with β-catenin to enter the nucleus from the cytoplasm, activating the downstream Wnt/β-catenin signaling pathway; ultimately, it is involved in the regulation of adhesion, migration, and tube formation of HUVECs. These results help to elucidate the mechanism of Kindlin-2 in the regulation of wound healing and provide a theoretical basis for further study of wound healing and abnormal healing.

## Discussion

In this study, we first demonstrated Kindlin-2 expression changes during the wound healing process. Such expression has previously been detected in many cell types, including fibroblasts, muscle cells, endothelial cells and epithelial cells, where it is concentrated within focal adhesions [24–26]. The findings of this study showed that Kindlin-2 knockdown inhibited wound healing and increased endothelial cell permeability in vivo, resulting in abnormal new vessel morphology.

Kindlin-2 is essential for embryonic development, as Kindlin-2-deficient embryonic stem cells have shown reduced adhesion to various substrates of the extracellular matrix, such as laminin-111, laminin-332, and fibronectin, which can lead to embryonic death [27]. Knockdown of Kindlin-2 has been reported to lead to a significant reduction in the invasive properties of cancer cells through nuclear factor kappa B (NF-κB)-dependent upregulation of the expression of matrix metallopeptidase (MMP)-9 and MMP-2 [28]. As a member of the adaptor protein family, Kindlin-2 can be recruited into the tail of the integrin β subunit and plays a role in binding with integrin, which can not only activate integrin together with talin but also recruit plaque adhesion protein to integrin and regulate the adhesion between cells and extracellular matrix mediated by integration [29,30]. This molecule can also regulate integrin-mediated signal transduction [31]. Kindlin-2 controls TGF-β signal transduction, and Sox9 expression regulates chondrogenesis [32]. Similarly, kindlin-2 interacts with EGFR and mediates EGF-induced breast cancer cell migration [33]. Moreover, Kindlin-2 can bind to β-catenin, activate the Wnt signaling pathway and promote tumor invasion and metastasis of hepatocellular carcinoma [34].

Canonical Wnt signaling components, such as β-catenin, have been implicated in epithelial tissue homeostasis by maintaining stem cell proliferation and migration, especially in the intestine, mammary gland, and skin [35,36]. The Wnt signaling pathway plays an important role in regulating the physiological functions of a variety of cells, including proliferation, repair and angiogenesis, and is involved in the regulation of tumorigenesis and development [37,38]. At present, there are at least three Wnt signaling pathways: the classic Wnt/β-catenin pathway and two nonclassical pathways [39]. After Wnt ligand stimulation, β-catenin translocates into the nucleus and combines with the TCF/LEF transcription factor to initiate Wnt-targeted gene transcription, which can effectively promote the division and chemotaxis of vascular endothelial cells [40]. The activation of the Wnt signaling pathway was shown to lead to the upregulation of VEGF expression [41]. We found that downregulation of kindlin-2 expression disrupted the localization of β-catenin, which inhibited its downstream molecules. Western blotting was used to detect the distribution of β-catenin in the nucleoplasm. We found that the translocation of β-catenin from the cytoplasm to the nucleus decreased significantly after kindlin-2 expression was downregulated. The expression of the β-catenin downstream molecules Met, c-jun, TCF-7 and c-Myc was also decreased. These results suggest that kindlin-2 may cooperate with β-catenin to enter the nucleus. The interaction between kindlin-2 and β-catenin is necessary for β-catenin to enter the nucleus.

In short, our research shows that kindlin-2 is expressed differently in the process of wound healing and can regulate angiogenesis during the wound healing process. Moreover, kindlin-2 influences the location of β-catenin, promoting its nuclear translocation in vascular endothelial cells and thus regulating the Wnt/β-catenin signaling pathway.

## Conclusion

The present study provides new insights into Kindlin-2 function in wound healing progression. Our data showed that downregulation of Kindlin-2 expression in mice suppresses the skin wound healing process with a decreased number of new blood vessels and collagen deposition. Depletion of Kindlin-2 impaired HUVEC spreading, migration and tube formation by targeting β-catenin, suggesting that Kindlin-2 is a novel therapeutic target for the management of skin wound healing. However, further studies are required to fully elucidate the molecular mechanisms and the specific binding sites between Kindlin-2 and β-catenin.

## Supplementary Material

Supplemental Material
