# Supplementary material for: Fermitin family homolog 2 (Kindlin-2) affects vascularization during the wound healing process by regulating the Wnt/β-catenin signaling pathway in vascular endothelial cells
Source: Bioengineered. 2021 Aug 2;12(1):4654–65. doi: 10.1080/21655979.2021.1957526 (PMC8806626; doi:10.1080/21655979.2021.1957526)
Supplement: Supplemental Material [file KBIE_A_1957526_SM9914.zip › supplfig.pdf]

Kindlin-2 shRNA1 group and Kindlin-2 shRNA2 group were detected by Western blots.

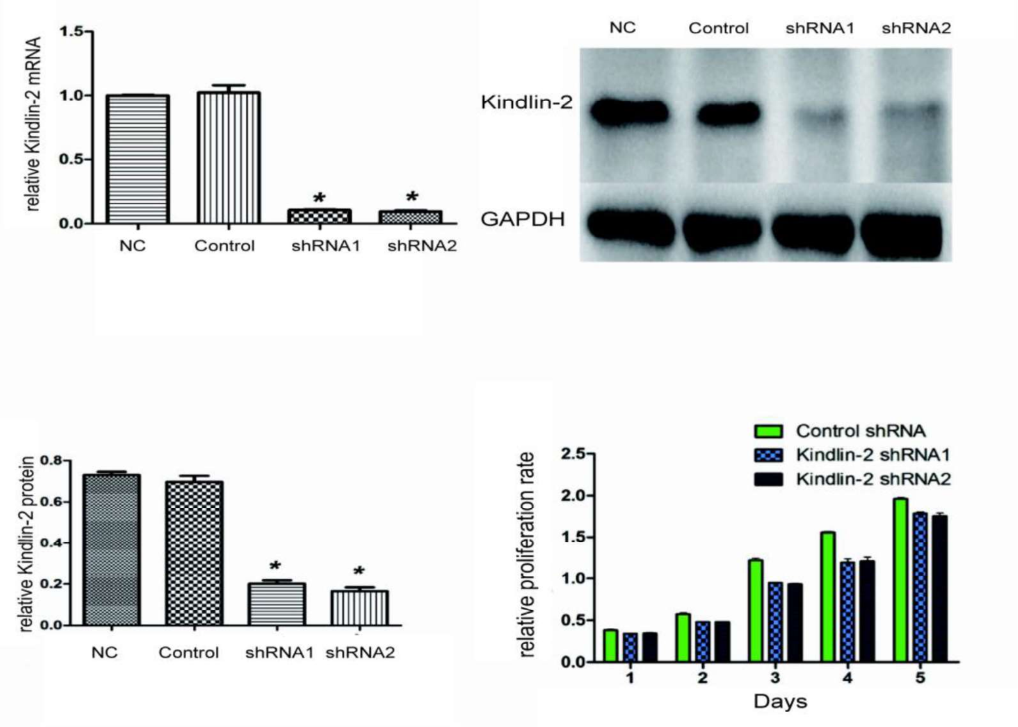

Supplemental Figure 1

a: RT-PCR detected the mRNA expression of kindlin-2 after kindlin-2 knockdown.

b: Western blot analysis of the protein expression of kindlin-2 after kindlin-2 knockdown.

c: The protein expression of kindlin-2 in each group was calculated by ImageJ.

d: CCK-8 assays detected the proliferation of cells in each group.
